# Patterns of Human Papillomavirus DNA and Antibody Positivity in Young Males and Females, Suggesting a Site-Specific Natural Course of Infection

**DOI:** 10.1371/journal.pone.0060696

**Published:** 2013-04-23

**Authors:** Henrike J. Vriend, Johannes A. Bogaards, Fiona R. M. van der Klis, Mirte Scherpenisse, Hein J. Boot, Audrey J. King, Marianne A. B. van der Sande, on behalf of the Medical Microbiological Laboratories, Municipal Health Services

**Affiliations:** 1 Centre for Infectious Disease Control, National Institute for Public Health and the Environment (RIVM), Bilthoven, The Netherlands; 2 Department of Internal Medicine, Division of Infectious Diseases, Tropical Medicine and AIDS, Center for Infection and Immunity Amsterdam (CINIMA), Academic Medical Center (AMC), Amsterdam, The Netherlands; 3 Department of Epidemiology and Biostatistics, VU University Medical Center, Amsterdam, The Netherlands; 4 Julius Center, University Medical Center, Utrecht, The Netherlands; Centers for Disease Control and Prevention, United States of America

## Abstract

**Background:**

To monitor the impact of human papillomavirus types 16 and 18 vaccine on HPV infection dynamics in the Netherlands, we started an ongoing study in sexually transmitted infection (STI) clinics in 2009. Here, we analyze baseline type-specific HPV DNA and HPV-specific antibody positivity rates.

**Methods:**

We enrolled 3569 men and women, 16–24 years of age, from 14 STI clinics, and estimated genital and anal HPV DNA and antibody positivity rates of 7 main carcinogenic HPV types. Generalized estimating equations regression analyses were applied to determine risk factors for, and associations between, type-specific HPV DNA and antibody positivity.

**Results:**

Genital HPV DNA positivity rates were higher in women than in men; anal HPV DNA was especially high in men who have sex with men (MSM). HPV antibody seropositivity rates were also highest in women and MSM. High-risk sexual behavior was predictive of both HPV DNA and antibody positivity. Despite a strong correlation in serological profiles for multiple HPV types, seropositivity was independently associated with homologous HPV DNA detection.

**Conclusions:**

HPV DNA and antibody positivity rates are higher in women and MSM than in heterosexual men, but their association is similar across gender. This suggests a site-specific natural course of infection.

## Introduction

Human papillomavirus (HPV) is a common sexually transmitted virus known for its causal relation to cervical cancer. There are more than 100 HPV genotypes, with more than 15 carcinogenic types [1], [2]. In many countries, HPV vaccination has been introduced in sexually naïve girls to prevent infections with HPV-16/-18, which are most commonly found in cervical cancers. It is not yet known what the impact of HPV vaccination will be on HPV dynamics in partially vaccinated populations. Monitoring of type-specific HPV prevalence in both vaccinated and nonvaccinated people is, therefore, of great importance.

HPV infection does not always induce an immune response that results in HPV-specific antibodies (Ab) [3], [4], [5]. Even if women are diagnosed with precancerous cervical lesions that test positive for HPV DNA, they might still be negative for serum HPV Ab [6]. Whether HPV infection will lead to seroconversion depends on several factors, such as specific HPV types, persistence of infection, HPV DNA viral load, and site of infection [3], [4], [7], [8], [9], [10], [11]. In contrast to natural infection, HPV vaccination induces an immune response with high concentrations of HPV Ab, by far exceeding the HPV Ab concentrations found in nonvaccinated populations [12]. In addition, studies showed that vaccination against HPV-16/-18 can result in cross-protection against phylogenetically related genotypes [13], [14]. Therefore, it is possible that vaccination may not only result in a decline in HPV-16/-18 prevalence, but also in a decline in phylogenetically related genotypes such as HPV-31, -33, and -45. Conversely, unwanted effects like type replacement, i.e., the potential for nonvaccine HPV types to occupy the vacated ecologic niches, can occur as a result of the elimination of HPV-16/-18 [15]. This hypothesis has neither been confirmed nor rejected by epidemiological studies. As a result of reduced exposure to HPV-16/-18, an effect can also be expected among nonvaccinated men and women [16], [17], [18], [19].

The aim of our study was to describe HPV DNA and HPV-specific Ab detection rates of women, men who have sex with women only (MSW), and men who have sex with men (MSM), all of whom were without benefit of HPV vaccination. Furthermore, we explored associations between homologous and heterologous pairs of HPV DNA and HPV Ab types. This description will serve as a baseline measurement to which we can compare future monitoring rounds on HPV dynamics within the Netherlands.

## Materials and Methods

### Ethics Statement

The medical ethics committee of the University of Utrecht, the Netherlands, confirmed in writing that they waived the need for separate ethical approval and the need for written consent. This anonymous study used serum already collected for routine STI consultation, therefore no additional invasive procedures were needed. All eligible participants were informed about the purpose of the study prior to the regular STI consultation and full information was provided about the samples to be collected and the additional questionnaire to be administered. Only participants who consented verbally with all conditions were included in the study.

### Study Population and Design

In 2009, the bivalent HPV vaccine was introduced in the Netherlands among 12- to 16-year-old girls. To monitor the effects of HPV-16/-18 vaccination on type-specific HPV dynamics in a young highly sexually active population, the PASSYON (PApillomavirus Surveillance among STI clinic YOungsters in the Netherlands) study was set up [20]. This biennial cross-sectional study includes 16- to 24-year-old male and female attendees of the sexually transmitted infection (STI) clinic. In 2009 and 2011, the first two rounds of this study took place in 14 STI clinics throughout the Netherlands; 10 STI clinics participated in both rounds.

A genital self-sample (vaginal or penile) of all participants was requested (sampling technique is described elsewhere [20]). In addition, MSM were requested for an anal sample as well. Procedures were similar in both rounds, however, in 2011, females and MSW also were invited to submit an anal sample. In addition, participation included: the use of serum already collected during routine consultation or collection of an extra blood sample if routine serum was not available; filling out a questionnaire; and permission to link the anonymous standardized data collected during the intake of the routine consultation with STI test results (e.g., *Chlamydia trachomatis*, *Neisseria gonorrhoea*, syphilis, HIV). The questionnaire covered sexual risk factors such as condom use, number of sex partners, and history of STI. Demographic data were also collected. Ethnicity was not recorded, but we asked the participants which population they belonged to, regardless of country of birth, and used their responses as proxies.

### HPV DNA detection

All swabs were suspended in 1 ml universal transport medium buffer and stored at −20°C until processing. After thawing, swabs were vortexed and 200 µL of the sample was spiked with phocine herpes virus-1. DNA was subsequently extracted using the MagnaPure platform (Total Nucleic Acid Isolation Kit, Roche) and eluted in 100 µL elution buffer. HPV-DNA was amplified using the SPF10 primer set according to the manufacturer's instructions (DDL Diagnostic Laboratory, the Netherlands [21]). HPV-specific amplicons were detected using the DNA enzyme-linked immunoassay (HPV-DEIA, DDL Diagnostic Laboratory, the Netherlands). Amplicons of HPV-positive samples were subsequently analyzed in Line probe assay (HPV-LiPA, DDL Diagnostic Laboratory, the Netherlands). In this report, only the seven carcinogenic HPV types, which can also be detected in the Ab detection assay, are reported (i.e., HPV-16, -18, -31, -33, -45, -52, -58). Only persons positive for one of the seven HPV-types are reported as being HPV positive.

### Serum antibody detection

Serum samples were stored at −80°C until analysis. HPV-specific serum antibodies against L1 virus-like particles (VLPs) for serotypes -16, -18, -31, -33, -45, -52 and -58 were assessed using a VLP-based multiplex immunoassay in which the VLPs were coupled to a set of seven distinct fluorescent microspheres. VLPs were kindly donated by GSK. Most samples were analysed with a 1/100 or 1/200 dilution, some in 1/2000 dilution. Serum samples were assumed to be antibody seropositive at cut-offs determined previously with this assay: 9, 13, 27, 11, 19, 14, 31 Luminex Units/millilitre (LU/ml) for HPV-16, -18, -31, -33, -45, -52, -58, respectively. The serological measurements and control steps have been described in more detail elsewhere [22].

### Statistical Analyses

Since no significant differences were observed in HPV DNA or Ab positivity rates between the first two rounds (2009 and 2011), we chose to combine these rounds in this study to gain more power. We included only HIV-negative persons having a urogenital DNA sample as well as a serum sample. Women who reported HPV vaccination were excluded in these baseline analyses. We did not exclude men who reported to be HPV vaccinated (n = 17), since Ab concentrations of HPV-16/-18 were not significantly different from those of persons who reported not to be HPV vaccinated, and since men are not (yet) vaccinated in the Netherlands for HPV, it was unlikely that they actually had been vaccinated. Type-specific positivity rates for the seven detectable HPV DNA and HPV Ab types were estimated, stratified by women, MSW, and MSM. Positivity rates were estimated by dividing the number of type-specific HPV DNA positives by the total number tested, with 95% confidence intervals (CI) based on Wilson's score method. The Student's t-test was used to compare positivity rates. When the number testing positive was below five the Fisher's exact test was used.

Generalized estimating equations (GEE) analyses were performed to assess independent risk factors associated with the presence of HPV DNA, and separate analyses were performed to asses independent risk factors associated with HPV Ab seropositivity. GEE models are marginal and take into account multiple measurements, making them highly suitable for estimating population-averaged risks associated with repeated measures [23]. GEE models estimate conditional log odds ratios (β_x_) between an independent risk factor (x) and a multivariate outcome variable, while assuming a particular correlation structure between the repeated measures, in this case multiple HPV types [24]. In assessing independent risk factors for the presence of HPV DNA and for HPV Ab seropositivity, we used an ‘exchangeable correlation’ structure with a common odds ratio alpha, which is appropriate when the association between pairs of HPV types can be assumed to be constant. Analyses were stratified by gender (i.e., women, MSW, MSM), since univariable analyses showed that all three outcome variables (genital HPV DNA, anal HPV DNA, and HPV Ab) differed significantly between women, MSW, and MSM. Multivariable analyses were performed by including the following variables into a GEE model: age, ethnicity, years sexually active, number of lifetime sex partners, number of sex partners in the last six months, condom use in the last six months with a steady partner, condom use in the last six months with a casual partner, a chlamydia or gonorrhea infection in the past, a current urogenital chlamydia infection, a current anogenital chlamydia or gonorrhea infection, passive anal sex in the past six months, and the seven different HPV genotypes. Subsequently, all nonsignificant (*p*≥0.05) variables were removed by backward selection.

GEE models were also used to estimate possible type-specific associations between the presence of HPV DNA and HPV Ab. These analyses were also stratified by gender, but could not be performed for MSM because of the small group size. For these analyses, we used a saturated model for the independent risk factors and started with a fully parameterized model for the pair-wise log odds ratios between all 14 genital HPV DNA and HPV Ab outcome variables. The starting log odds ratio model was composed of 14×13/2 = 91 linear parameter combinations, one for each unique binary pair of type-specific DNA and/or Ab of the seven HPV types. All nonsignificant parameters were subsequently deleted from the model using Bonferroni-corrected *p* values based on Wald tests, and this procedure was repeated until all remaining parameters were significant. Final estimates were obtained after orthonormal transformation of the log odds ratio estimation matrix. In modelling the association between pairs of responses, we made use of the alternating logistic regression algorithm of the SAS GENMOD procedure [25].

## Results

### Characteristics study population

In 2009 and 2011, 1637 and 1932 participants, respectively, were tested for a genital HPV infection. Of these 3569 participants, 3439 (96%) were also tested for HPV Ab. After exclusion of 6 HIV-positive persons and 105 self-reported HPV-vaccinated women, a study population of 3328 participants remained, of whom two-thirds were females (n = 2233). A subgroup of 118 (5%) women, 56 (6%) MSW, and 124 (72%) MSM also had a test result for anal HPV DNA infection.

Characteristics stratified for women, MSW, and MSM are given in Table 1. The median age of the 3328 participants was 21 years (IQR: 20–23 years). Of all men, 16% (n = 173) reported having sex with men. Sexual behavioral factors showed a median age of first intercourse of 16 years (IQR: 15–17 years) and a median number of lifetime partners of 7 (IQR: 4–12) (6, 9, and 12 lifetime partners for women, MSW, and MSM, respectively). The percentage of persons with a current or past anogenital chlamydia or gonorrhoea infection is given in Table 2. Among women and MSW, 14% had a current urogenital chlamydia and 2% had a current gonorrhea infection. For MSM this was 5% and 4%, respectively. A current anal chlamydia and/or gonorrhea infection was reported for 1% of women and 14% of MSM. No anal infections were seen in MSW.

**Table 1:** Demographics and characteristics of the study population: women, MSW, and MSM.

		Female		MSW		MSM		Total	
		N = 2233		N = 922		N = 173		N = 3328	
		n	*(%)*	n	*(%)*	n	*(%)*	n	*(%)*
**Year of participation**									
	2009	1069	*(47.9)*	414	*(44.9)*	68	*(39.3)*	1551	*(46.6)*
	2011	1164	*(52.1)*	508	*(55.1)*	105	*(60.7)*	1777	*(53.4)*
**Age**									
	16–18 years	249	*(11.2)*	61	*(6.6)*	17	*(9.8)*	327	*(9.8)*
	19–21 years	974	*(43.6)*	351	*(38.1)*	71	*(41.0)*	1396	*(41.9)*
	22–24 years	1010	*(45.2)*	510	*(55.3)*	85	*(49.1)*	1605	*(48.2)*
**Ethnicity**									
	Dutch	1925	*(86.2)*	741	*(80.4)*	137	*(79.2)*	2803	*(84.2)*
	Non-Dutch	308	*(13.8)*	181	*(19.6)*	36	*(20.8)*	525	*(15.8)*
**Educational level** *									
	Low	512	*(22.9)*	281	*(30.5)*	57	*(32.9)*	850	*(25.5)*
	High	1655	*(74.1)*	605	*(65.6)*	109	*(63.0)*	2369	*(71.2)*
	Unknown	66	*(3.0)*	36	*(3.9)*	7	*(4.0)*	109	*(3.3)*
**Years sexual active**									
	0–2 years	411	*(18.4)*	97	*(10.5)*	32	*(18.5)*	540	*(16.2)*
	3–4 years	576	*(25.8)*	224	*(24.3)*	47	*(27.2)*	847	*(25.5)*
	≥5 years	1179	*(52.8)*	562	*(61.0)*	87	*(50.3)*	1828	*(54.9)*
	Unknown	67	*(3.0)*	39	*(4.2)*	7	*(4.0)*	113	*(3.4)*
**Steady or casual sex partner**									
	No partner	1009	*(45.2)*	348	*(37.7)*	71	*(41.0)*	1428	*(42.9)*
	Steady partner	878	*(39.3)*	409	*(44.4)*	61	*(35.3)*	1348	*(40.5)*
	Casual partner	237	*(10.6)*	99	*(10.7)*	21	*(12.1)*	357	*(10.7)*
	Steady and casual partner	52	*(2.3)*	28	*(3.0)*	14	*(8.1)*	94	*(2.8)*
	Unknown	57	*(2.6)*	38	*(4.1)*	6	*(3.5)*	101	*(3.0)*
**Condom use steady partner, past 6 months**									
	Inconsistent condom use	726	*(32.5)*	337	*(36.6)*	45	*(26.0)*	1108	*(33.3)*
	Consistent condom use	201	*(9.0)*	98	*(10.6)*	30	*(17.3)*	329	*(9.9)*
	No steady partner	1251	*(56.0)*	449	*(48.7)*	92	*(53.2)*	1792	*(53.8)*
	Unknown	55	*(2.5)*	38	*(4.1)*	6	*(3.5)*	99	*(3.0)*
**Condom use casual partner, past 6 months**									
	Inconsistent condom use	728	*(32.6)*	349	*(37.9)*	31	*(17.9)*	1108	*(33.3)*
	Consistent condom use	834	*(37.3)*	317	*(34.4)*	117	*(67.6)*	1268	*(38.1)*
	No casual partner	620	*(27.8)*	220	*(23.9)*	19	*(11.0)*	859	*(25.8)*
	Unknown	51	*(2.3)*	36	*(3.9)*	6	*(3.5)*	93	*(2.8)*
**Number of sex partners, lifetime**									
	1–4 partners	762	*(34.1)*	187	*(20.3)*	29	*(16.8)*	978	*(29.4)*
	5–9 partners	804	*(36.0)*	249	*(27.0)*	29	*(16.8)*	1082	*(32.5)*
	≥10 partners	612	*(27.4)*	442	*(47.9)*	113	*(65.3)*	1167	*(35.1)*
	Unknown	55	*(2.5)*	44	*(4.8)*	2	*(1.2)*	101	*(3.0)*
**Number of sex partners, past 6 months**									
	0–1 partners	806	*(36.1)*	232	*(25.2)*	28	*(16.2)*	1066	*(32.0)*
	2–3 partners	1048	*(46.9)*	379	*(41.1)*	62	*(35.8)*	1489	*(44.7)*
	≥4 partners	375	*(16.8)*	310	*(33.6)*	83	*(48.0)*	768	*(23.1)*
	Unknown	4	*(0.2)*	1	*(0.1)*	0	*(0.0)*	5	*(0.2)*
**Passive anal intercourse, past 6 months**									
	No	1938	*(86.8)*	917	*(99.5)*	44	*(25.4)*	2899	*(87.1)*
	Yes	253	*(11.3)*	5	*(0.5)*	129	*(74.6)*	387	*(11.6)*
	Unknown	42	*(1.9)*	0	*(0.0)*	0	*(0.0)*	42	*(1.3)*

* High educational level includes university of science and university of professional education, low educational level includes all other forms of education.

MSM = men who have sex with men; MSW = men who have sex with women only.

**Table 2:** Past and current (ano)genital chlamydia and gonorrhea infections: women, MSW, and MSM.

		Female		MSW		MSM		Total	
		N = 2233		N = 922		N = 173		N = 3328	
		n	*(%)*	n	*(%)*	n	*(%)*	n	*(%)*
**Chlamydia or gonorrhea ever**									
	No	1424	*(63.8)*	568	*(61.6)*	107	*(61.8)*	2099	*(63.1)*
	Yes	420	*(18.8)*	127	*(13.8)*	41	*(23.7)*	588	*(17.7)*
	Never tested before	326	*(14.6)*	190	*(20.6)*	16	*(9.2)*	532	*(16.0)*
	Unknown	63	*(2.8)*	37	*(4.0)*	9	*(5.2)*	109	*(3.3)*
**Current urogenital chlamydia infection**									
	No	1917	*(85.8)*	792	*(85.9)*	164	*(94.8)*	2873	*(86.3)*
	Yes	307	*(13.7)*	122	*(13.2)*	8	*(4.6)*	437	*(13.1)*
	Unknown/not tested	9	*(0.4)*	8	*(0.9)*	1	*(0.6)*	18	*(0.5)*
**Current urogenital gonorrhea infection**									
	No	2194	*(98.3)*	897	*(97.3)*	164	*(94.8)*	3255	*(97.8)*
	Yes	32	*(1.4)*	17	*(1.8)*	7	*(4.0)*	56	*(1.7)*
	Unknown/not tested	7	*(0.3)*	8	*(0.9)*	2	*(1.2)*	17	*(0.5)*
**Current anal chlamydia infection**									
	No	96	*(4.3)*	5	*(0.5)*	121	*(69.9)*	222	*(6.7)*
	Yes	24	*(1.1)*	0	*(0.0)*	16	*(9.2)*	40	*(1.2)*
	Unknown/not tested	2113	*(94.6)*	917	*(99.5)*	36	*(20.8)*	3066	*(92.1)*
**Current anal gonorrhea infection**									
	No	340	*(15.2)*	8	*(0.9)*	141	*(81.5)*	489	*(14.7)*
	Yes	7	*(0.3)*	0	*(0.0)*	13	*(7.5)*	20	*(0.6)*
	Unknown/not tested	1886	*(84.5)*	914	*(99.1)*	19	*(11.0)*	2819	*(84.7)*

MSM = men who have sex with men; MSW = men who have sex with women only.

### HPV DNA positivity and HPV-specific antibody seropositivity

**Figure 1:**
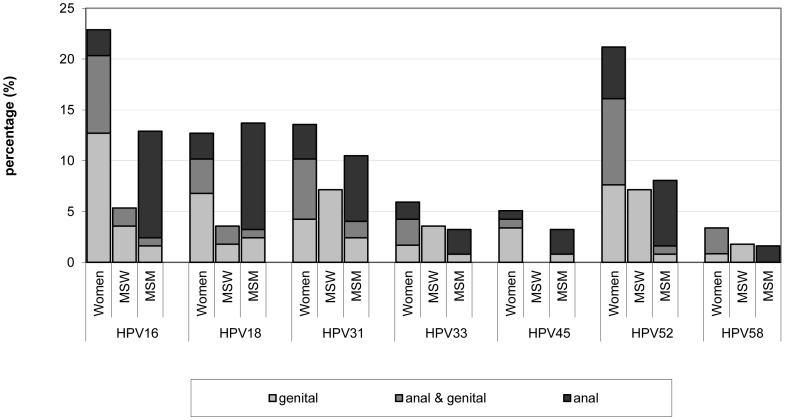
Positivity rate for genital and anal DNA detection in women, MSW and MSM. Black gives the positivity rate for persons with a HPV DNA detection only at the genitals, dark grey gives the positivity rate for persons with an HPV DNA infection genital as well as anal, and light grey are the positivity rates when persons are only detected with HPV DNA anally. The figures includes women (N = 118), MSW (N = 56) and MSM (N = 124) of whom a genital and an anal sample were available. MSW =  men who have sex with women only; MSM = men who have sex with men.

Women had the highest genital HPV DNA positivity rate (45%) with the highest type-specific rate for HPV-16 (17% of all women). Among men, MSM had lower positivity rates for genital (penile) DNA than MSW, 16% versus 26%, respectively. Although low genital HPV DNA positivity rates were seen in MSM, anal HPV DNA positivity rates were high. Within the subgroups that also provided anal samples (124 MSM, 118 women, and 56 MSW), similar anal HPV DNA positivity rates were seen in MSM (33%) and women (32%), whereas anal HPV DNA positivity was rare in MSW (4%) (Figure 1). HPV seropositivity was highest in women (49%), followed by MSM (34%) and MSW (19%).

**Figure 2:**
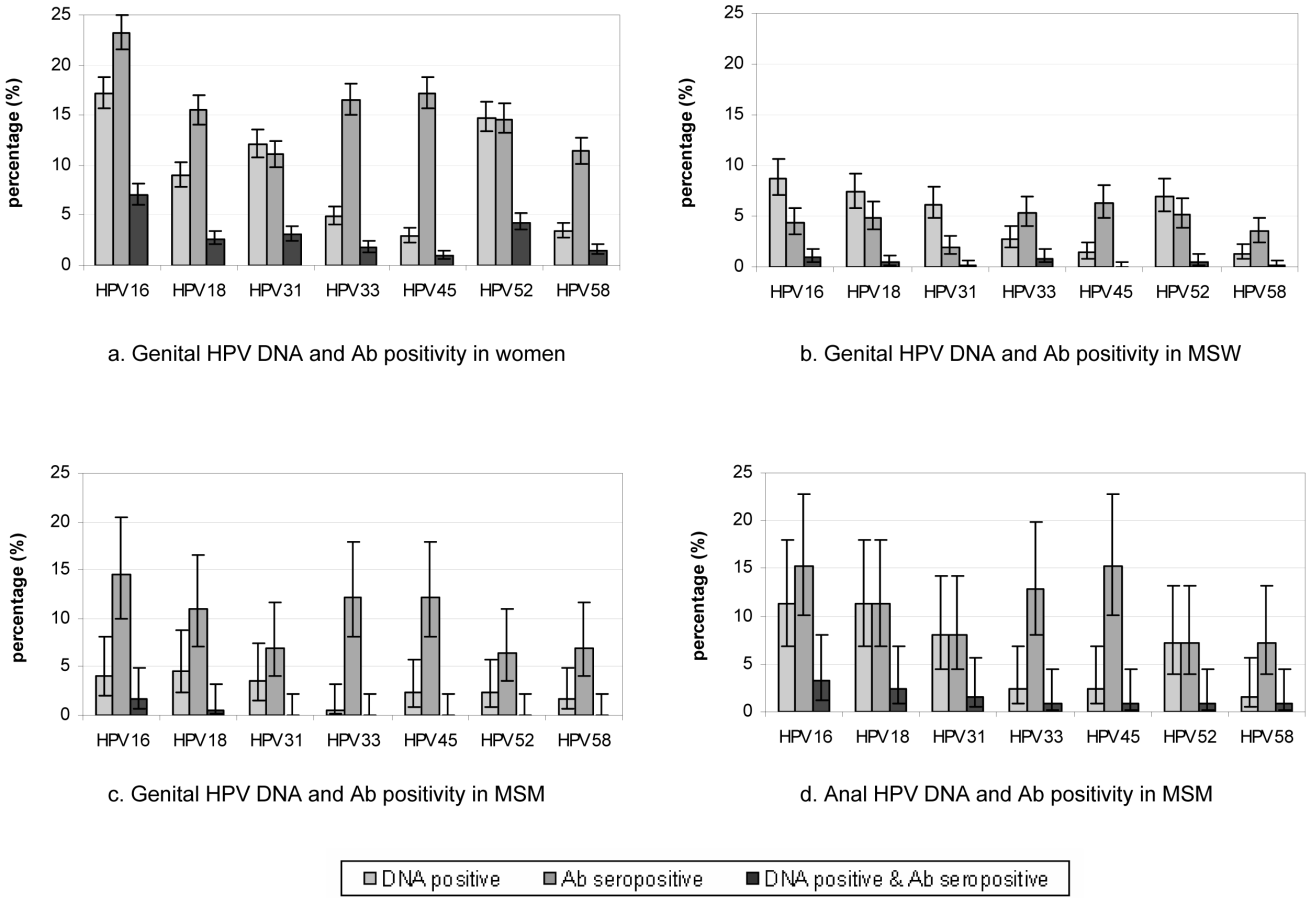
Genital (and anal) HPV DNA and HPV-specific antibody seropositivity in women, MSW, and MSM. Figure 2a–c gives the genital HPV DNA (light grey), HPV-specific antibody seropositivity (dark grey), as well as the percentage of persons detected with genital HPV DNA as well as HPV antibodies (black) with 95% confidence intervals in women (N = 2233), MSW (N = 922), and MSM (N = 173) per HPV genotype. Figure 2d gives the anal HPV DNA (light grey), HPV-specific antibody seropositivity (dark grey) as well as the percentage of persons detected with anal HPV DNA as well as HPV antibodies (black) with 95% confidence intervals in MSM (N = 124). Ab = antibody; MSM = men who have sex with men; MSW = men who have sex with women only.

Comparing HPV DNA positivity and HPV Ab seropositivity rates by gender, the main differences were the lower positivity rates for HPV Ab -16, -18, -31, and -52 compared to homologous HPV DNA positivity rates in MSW (Figure 2a–c). Women and MSM, however, had generally higher positivity rates for HPV Ab in comparison to homologous HPV DNA positivity rates. When comparing anal and genital infections in MSM, the anal HPV DNA positivity rates were higher than the genital HPV DNA positivity rates, although not significant (p = 0.25) (Figure 2c–d). Subsequently, the percentage of simultaneous detection of HPV DNA and homologous HPV Ab increased. The difference between anal HPV DNA positivity and HPV Ab seropositivity in MSM was similar to the genital rates observed in women. When observing women and MSM with both a genital as well as an anal sample, differences were seen for HPV Ab seropositivity among those infected with HPV DNA at one anatomical site only. Although numbers were small, women tended to be more often HPV Ab seropositive when only a genital HPV DNA infection was present compared to MSM (45% versus 18%, respectively (p = 0.16)). Whereas, HPV Ab seropositivity rates when only an anal HPV DNA infection was present were quite similar between women and MSM (29% versus 21%, respectively (p = 0.71)).

Simultaneous detection of type-specific HPV DNA and homologous HPV Ab genotype was most frequent for genotype 16 among all genders and sites. From Figure 2a–b, the odds ratio for HPV-16 Ab seropositivity together with HPV-16 DNA positivity is directly estimated at 2.9 for women and at 3.3 for MSW. However, given the presence of HPV-16 DNA, the probability of testing positive for HPV-16 Ab is much higher for women than for MSW, 41% versus 11%, respectively (p<0.01).

### Determinants of HPV DNA positivity and HPV-specific antibody seropositivity

Multivariable analyses resulted in different risk factors for genital HPV DNA positivity, anal HPV DNA positivity, and HPV Ab seropositivity. Most noticeable was the significant association of ethnicity with HPV Ab seropositivity for women as well as MSM, whereas ethnicity was not significantly associated with HPV DNA positivity. Persons who reported they were non-Dutch were more likely to be HPV Ab seropositive. Furthermore, in women the site of the current chlamydia infection corresponded with the site of the associated HPV DNA positivity: a genital chlamydia infection was associated with genital HPV DNA infection and an anal chlamydia infection with an anal HPV DNA infection. In MSM, reporting passive anal sex in the past six months was associated with HPV Ab seropositivity. All significant risk factors resulting from the multivariable GEE analysis are shown in Table 3, 4 and 5.

**Table 3:** Adjusted odds ratio and 95% confidence interval for the detection of genital and anal HPV DNA and HPV antibodies using multilevel analyses (generalized estimating equations [GEE)]) in women.

		Genital HPV DNA		Anal HPV DNA		HPV antibodies	
		AOR	(95% CI)	AOR	(95% CI)	AOR	(95% CI)
**Ethnicity**							
	Dutch	NS		NS		Reference	
	Non-Dutch					1.46	(1.19–1.78)
**Years sexual active**							
	0–2 years	NS		NS		Reference	
	3–4 years					0.95	(0.73–1.22)
	≥5 years					1.20	(0.95–1.51)
**Number of sex partners, lifetime**							
	1–4 partners	Reference		Reference		Reference	
	5–9 partners	1.37	(1.15–1.62)	0.31	(0.10–0.93)	1.40	(1.15–1.70)
	≥10 partners	2.02	(1.70–2.40)	0.94	(0.32–2.76)	2.23	(1.82–2.74)
**Condom use steady partner, past 6 months**							
	Inconsistent condom use	NS		NS		Reference	
	Consistent condom use					0.79	(0.59–1.04)
	No steady partner					0.82	(0.70–0.96)
**Condom use casual partner, past 6 months**							
	Inconsistent condom use	Reference		NS		NS	
	Consistent condom use	0.94	(0.82–1.08)				
	No casual partner	0.66	(0.56–0.79)				
**History of chlamydia or gonorrhea**							
	No	Reference		Reference		Reference	
	Yes	1.29	(1.10–1.51)	4.52	(1.46–14.02)	1.83	(1.53–2.19)
	Never tested before	1.08	(0.90–1.30)	1.32	(0.52–3.39)	0.80	(0.63–1.03)
**Current urogenital chlamydia infection**							
	No	Reference		NS		NS	
	Yes	1.26	(1.06–1.50)				
**Current anal chlamydia/gonorrhea infection**							
	No	Reference		Reference		Reference	
	Yes	0.50	(0.28–0.91)	3.45	(1.01–11.73)	1.33	(0.76–2.36)
	Not tested	0.77	(0.66–0.91)	0.52	(0.25–1.10)	0.65	(0.54–0.79)
**HPV genotype**							
	HPV16	Reference		Reference		Reference	
	HPV18	0.49	(0.41–0.59)	0.54	(0.21–1.42)	0.59	(0.51–0.68)
	HPV31	0.65	(0.55–0.77)	0.81	(0.37–1.80)	0.38	(0.32–0.45)
	HPV33	0.24	(0.19–0.30)	0.21	(0.06–0.77)	0.63	(0.55–0.72)
	HPV45	0.15	(0.11–0.19)	0.14	(0.03–0.60)	0.66	(0.58–0.76)
	HPV52	0.82	(0.70–0.97)	1.44	(0.69–3.03)	0.54	(0.47–0.62)
	HPV58	0.17	(0.13–0.22)	0.22	(0.05–0.86)	0.40	(0.34–0.46)
**Odds of having one HPV genotype when having another HPV genotype within an individual (Alpha)**							
		1.54	(1.35–1.76)	1.81	(1.01–3.23)	4.34	(3.79–4.98)

The following variables were included into the crude GEE model: age, ethnicity, years sexually active, number of lifetime sex partners, number of sex partners in the last six months, condom use in the last six months with a steady partner, condom use in the last six months with a casual partner, a chlamydia or gonorrhea in the past, a current urogenital chlamydia infection, a current anal chlamydia or gonorrhea infection, passive anal intercourse in the past six months, and the seven different HPV genotypes.

AOR = adjusted odds ratio; CI = confidence interval; NI  =  not included in the multivariable model; NS  =  not significant in multivariable model.

**Table 4:** Adjusted odds ratio and 95% confidence interval for the detection of genital and anal HPV DNA and HPV antibodies using multilevel analyses (generalized estimating equations [GEE)]) in men who have sex with women only (MSW).

		Genital HPV DNA		Anal HPV DNA*		HPV antibodies	
		AOR	(95% CI)	AOR	(95% CI)	AOR	(95% CI)
**Age (y)**							
	16–18	Reference				Reference	
	19–21	1.42	(0.67–2.99)			1.99	(0.93–4.27)
	22–24	2.02	(0.98–4.19)			2.19	(1.08–4.41)
**Number of sex partners, lifetime**							
	1–4 partners	Reference				NS	
	5–9 partners	1.39	(0.85–2.29)				
	≥10 partners	2.23	(1.45–3.43)				
**Condom use steady partner, past 6 months**							
	Inconsistent condom use	Reference				NS	
	Consistent condom use	0.33	(0.18–0.60)				
	No steady partner	0.74	(0.56–0.98)				
**History of chlamydia or gonorrhea**							
	No	Reference				NS	
	Yes	1.49	(1.05–2.12)				
	Never tested before	0.86	(0.58–1.27)				
**HPV genotype**							
	HPV16	Reference				Reference	
	HPV18	0.86	(0.62–1.21)			1.10	(0.73–1.66)
	HPV31	0.68	(0.47–0.97)			0.41	(0.24–0.71)
	HPV33	0.29	(0.19–0.47)			1.27	(0.86–1.86)
	HPV45	0.16	(0.09–0.28)			1.44	(0.95–2.18)
	HPV52	0.78	(0.55–1.10)			1.14	(0.78–1.66)
	HPV58	0.14	(0.08–0.26)			0.77	(0.50–1.17)
**Odds of having one HPV genotype when having another HPV genotype within an individual (Alpha)**							
		2.18	(1.46–3.24)			8.73	(5.94–12.83)

The following variables were included into the crude GEE model: age, ethnicity, years sexually active, number of lifetime sex partners, number of sex partners in the last six months, condom use in the last six months with a steady partner, condom use in the last six months with a casual partner, a chlamydia or gonorrhea in the past, a current urogenital chlamydia infection, passive anal intercourse in the past six months, and the seven different HPV genotypes.

* The model for anal HPV DNA could not be run for MSW due to small numbers.

AOR = adjusted odds ratio; CI = confidence interval; NI  =  not included in the multivariable model; NS  =  not significant in multivariable model.

**Table 5:** Adjusted odds ratio and 95% confidence interval for the detection of genital and anal HPV DNA and HPV antibodies using multilevel analyses (generalized estimating equations [GEE)]) in men who have sex with men (MSM).

		Genital HPV DNA*		Anal HPV DNA		HPV antibodies	
		AOR	(95% CI)	AOR	(95% CI)	AOR	(95% CI)
**Ethnicity**							
	Dutch			NS		Reference	
	Non-Dutch					2.65	(1.29–5.46)
**Years sexual active**							
	0–2 years			Reference		NS	
	3–4 years			6.23	(1.51–25.65)		
	≥5 years			7.74	(1.88–31.92)		
**Number of sex partners, lifetime**							
	1–4 partners			NS		Reference	
	5–9 partners					0.95	(0.24–3.78)
	≥10 partners					2.96	(1.02–8.57)
**Condom use casual partner, past 6 months**							
	Inconsistent condom use			NS		Reference	
	Consistent condom use					1.10	(0.39–3.10)
	No casual partner					5.38	(1.45–20.02)
**Passive anal intercourse, past 6 months**							
	No			NS		Reference	
	Yes					2.67	(1.25–5.72)
**HPV genotype**							
	HPV16			Reference		Reference	
	HPV18			1.00	(0.45–2.20)	0.65	(0.38–1.12)
	HPV31			0.68	(0.28–1.64)	0.39	(0.20–0.76)
	HPV33			0.19	(0.06–0.65)	0.81	(0.46–1.43)
	HPV45			0.19	(0.05–0.71)	0.75	(0.44–1.29)
	HPV52			0.53	(0.20–1.40)	0.38	(0.20–0.72)
	HPV58			0.13	(0.03–0.58)	0.34	(0.17–0.69)
**Odds of having one HPV genotype when having another HPV genotype within an individual (Alpha)**							
				1.79	(0.94–3.43)	8.12	(4.43–14.91)

The following variables were included into the crude GEE model: age, ethnicity, years sexually active, number of lifetime sex partners, number of sex partners in the last six months, condom use in the last six months with a steady partner, condom use in the last six months with a casual partner, a chlamydia or gonorrhea in the past, a current urogenital chlamydia infection, a current anal chlamydia or gonorrhea infection, passive anal intercourse in the past six months, and the seven different HPV genotypes.

* The model for genital HPV DNA could not be run for MSM due to small numbers.

AOR = adjusted odds ratio; CI = confidence interval; NI  =  not included in the multivariable model; NS  =  not significant in multivariable model.

### Association between genital HPV DNA and HPV-specific antibody genotypes

The log odds ratio model for genital HPV DNA positivity and HPV Ab seropositivity could be reduced from a total of 91 parameters to as few as 7 independent parameters in women. Table 6 shows the resulting estimates of the common odds ratios for any pair of the following: (1) homologous HPV DNA and HPV Ab genotypes; (2) HPV DNA genotypes; (3) HPV Ab genotypes; and (4) heterologous DNA and Ab genotypes. In addition to these associations, we found significantly increased odds ratios for DNA and Ab of HPV-58; DNA of HPV-31 and HPV-33; and Ab detection for a subset of genotypes (i.e., HPV-33, -45, -52, -58). For example, seropositivity for HPV-33 Ab in combination with HPV-45 Ab is a frequently occurring test result, as well as seropositivity for HPV-33 Ab with HPV-52 Ab. For MSW, the number of independent parameters that could be estimated was smaller than for women, which is in line with the smaller sample size and lower positivity rate in MSW as compared to women. Associations were similar between the sexes (Table 6), except that a much stronger association was observed between pairs of HPV Ab genotypes in MSW compared with women.

**Table 6:** Association (odds ratio and 95% confidence interval) between genital HPV DNA and HPV-specific antibody genotypes, for women and MSW.

	Women		MSW	
	OR	(95% CI)	OR	(95% CI)
Any pair of homologous HPV DNA and HPV Ab genotypes	2.55	(2.19–2.96)	2.02	(1.28–3.20)
Any pair of HPV DNA genotypes	1.54	(1.35–1.76)	2.07	(1.36–3.15)
Any pair of HPV Ab genotypes	4.14	(3.61–4.76)	9.07	(6.01–13.69)
Any pair of heterologous DNA and Ab genotypes	1.21	(1.09–1.33)	1.01	(0.66–1.53)

Ab = antibody; OR = odds ratio; CI = confidence interval; MSW = men who have sex with women only.

The results of the log odds ratio model provided a justification for the assumption of an ‘exchangeable correlation’ structure in assessing independent risk factors for the presence of HPV DNA and for HPV Ab seropositivity. The associations between HPV DNA genotypes or between HPV Ab genotypes estimated by the log odds ratio model corresponded well with the alphas estimated in the multivariable GEE analyses (Table 3, 4 and 5). Note that the common odds ratios estimated by assuming an exchangeable correlation structure are slightly higher (with exception of the odds ratio for HPV Ab in MSW), because these incorporate the small elevations related to certain HPV types in the log odds ratio model. Having verified the assumptions underlying an exchangeable correlation structure, we then compared the associations between HPV DNA genotypes or HPV Ab genotypes in MSM and those in women and in MSW. We found that MSM have similar associations between anal HPV DNA genotypes as women (Table 3 and 5). Associations between HPV Ab genotypes of MSM overlap with those of women and MSW when taking the 95% confidence intervals into account.

## Discussion

Positivity rates were high in this young and sexually active population, without the benefit of HPV vaccination. HPV DNA positivity and HPV Ab seropositivity were higher in women than in MSW, with MSM in between, but the association between detection of type-specific DNA and serum Ab was similar across gender. The PASSYON study sheds new light on differences in the natural history of HPV infection between men and women, indirectly providing evidence for a site-specific natural course of infection.

In women, HPV DNA positivity rates were lower for each HPV genotype than HPV Ab seropositivity rates, whereas in MSW, type-specific HPV DNA positivity rates were higher than HPV Ab seropositivity rates with the exceptions of HPV Ab-33, -45, and -58. These specific Ab types had high positivity rates in all genders, which was confirmed by the results of the GEE analyses. Significantly higher association between Ab-33 and -45 and between Ab-33 and -58 was found, meaning that when an individual is positive for one of these Ab types, he or she is also frequently found positive for the other type. The gender differences we observed in HPV DNA positivity and HPV Ab seropositivity may be related to the type of tissue being infected. The dry keratinized tissue of the penis is much harder for the virus to infect than the soft mucosal tissue of the vagina or the anus, therefore men are less likely than women to develop a humoral response [8], [9], [10], [11]. Results in MSM support the hypothesis that tissue could play an important role in HPV Ab seropositivity, since high HPV Ab seropositivity rates in combination with high anal HPV DNA positivity rates were found in MSM, whereas genital HPV DNA positivity was low. The ratios between HPV Ab and anal HPV DNA positivity rates in MSM were similar to the ratios of HPV Ab and vaginal/anal HPV DNA positivity rates in women. To examine whether gender-specific factors other than anatomical site could influence seroconversion, we also compared the HPV Ab seropositivity rates within women and MSM who provided both genital and anal samples. Women with only a genital HPV DNA infection were more often seropositive for the homologous HPV Ab serotype as compared to MSM with only a penile HPV DNA infection, whereas HPV Ab seropositivity rates were comparable for women and MSM with only an anal HPV DNA infection. These findings support the hypothesis that penile HPV infections will result less often in seroconversion than vaginal or anal HPV infections. However, our study was not adequately powered to detect differences in seropositivity rate between women and MSM who were infected at one anatomical site only, and we cannot rule out the possibility that additional factors (e.g. genetic, behavioural) might contribute to gender-specific differences in seroconversion. We can only conjecture that anatomical site is a determinant in the natural course of HPV infection. Obviously, a cross-sectional design is inherently limited in drawing conclusions about dynamic associations and it would be worthwhile to further investigate the relation between site-specific HPV infection and the humoral immune response in a longitudinal study.

Multivariable GEE analyses helped clarify risk factors for HPV positivity. One risk factor for a HPV DNA infection in women was a current chlamydia infection. The site-specific relation between a chlamydia infection and an HPV infection was noticeable: a current genital chlamydia infection was associated with genital HPV DNA positivity and a current anal chlamydia infection with anal HPV DNA positivity. The underlying mechanism could be less efficient clearance because of this concurrent infection, resulting in a persistent infection, or easier invasion into the body. There were also behavioral factors, such as reported number of lifetime sex partners, associated with HPV DNA positivity and HPV Ab seropositivity. These results are in line with previous studies [11], [26], [27], [28], [29], [30].

The added value of GEE analyses is the possibility to include all type-specific HPV DNA and HPV Ab outcomes in one model, thereby making optimal use of the available data. In addition, including both HPV DNA and HPV Ab test results in one model gave us the option to estimate associations between homologous and heterologous pairs of HPV DNA and HPV Ab genotypes. Despite the difference in HPV DNA positivity and HPV Ab seropositivity between women and MSW, no significant gender difference was seen in the association between a homologous or heterologous pair of HPV DNA and HPV Ab types. Equal odds ratios for the associations between HPV DNA positivity and HPV Ab seropositivity in women and MSW do not imply that the natural course of the infection would be similar between the sexes. As shown, women have a higher probability to be seropositive when HPV DNA positive then MSW. A higher degree of persistence could be an explanation for the higher seropositivity in women. Furthermore, a higher association between any two HPV Ab genotypes in MSW was observed as compared to women. This higher association in MSW could be due to the less frequent occurrence of seroconversion. It is conceivable that characteristics related to seroconversion are restricted to specific persons such that, on a population level, it will lead to a stronger clustering of seropositivity in MSW than in women, where seroconversion is more common.

We also found type-specific HPV DNA and HPV Ab associations and found some associations between homologous HPV DNA and HPV Ab types, and between two heterologous HPV DNA types.

These associations could have occurred because of the high level of multiple HPV infections. However, the HPV types tested in the VLP-MIA are phylogenetically related to each other, and belong to the alpha-7 species (HPV-18, -45) and alpha-9 species (HPV-16, -31, -33, -52, -58) [31]. Since a high percentage of homology is found between HPV types of the same species, cross-reactive HPV-specific antibodies could account for the type-specific associations.

To summarize, the higher HPV DNA and Ab positivity rates in women and MSM as compared to heterosexual men, and the similar HPV DNA and Ab associations across gender suggest a site-specific natural course of infection. These findings in combination with the determinants for HPV DNA positivity and HPV Ab seropositivity can help us interpret the results of future rounds with more accuracy and will enable us to detect changes in HPV infection dynamics, both of vaccine and nonvaccine types.
